# Correction: Inhibitors of Rho kinases (ROCK) induce multiple mitotic defects and synthetic lethality in BRCA2-deficient cells

**DOI:** 10.7554/eLife.94414

**Published:** 2023-11-14

**Authors:** Julieta Martino, Sebastián Omar Siri, Nicolás Luis Calzetta, Natalia Soledad Paviolo, Cintia Garro, Maria F Pansa, Sofía Carbajosa, Aaron C Brown, José Luis Bocco, Israel Gloger, Gerard Drewes, Kevin P Madauss, Gastón Soria, Vanesa Gottifredi

**Keywords:** Other

Martino J, Siri SO, Calzetta NL, Paviolo NS, Garro C, Pansa MF, Carbajosa S, Brown AC, Bocco JL, Gloger I, Drewes G, Madauss KP, Soria G, Gottifredi V. 2023. Inhibitors of Rho kinases (ROCK) induce multiple mitotic defects and synthetic lethality in BRCA2-deficient cells. *eLife* **12**:e80254. doi: 10.7554/eLife.80254.

Published 19 April 2023

We were alerted by a PubPeer thread about a partial overlap of two representative images that reveals a duplication which affects two fields in panels 8B (PEO4 /SiROCK 1/2) and 8E (PEO4 SiLUC Field #1). https://pubpeer.com/publications/7725B88BE4284DAD6AED99752AB92F. The mistake arose when incorrectly using a PEO4 /SiROCK 1/2 image belonging to panel 8B to generate a PEO4 siLUC Field #1 image from panel 8E. We have identified the correct image and used it to generate a corrected PEO4 SiLUC Field #1 image in Figure 8E. This mistake does not affect the quantifications associated with Figure 8 B and E and the conclusions of the manuscript. The corrected Figure 8E, including the correct PEO4 siLUC Field #1 is appended below.

[Corrected Figure 8]

**Figure 1:**
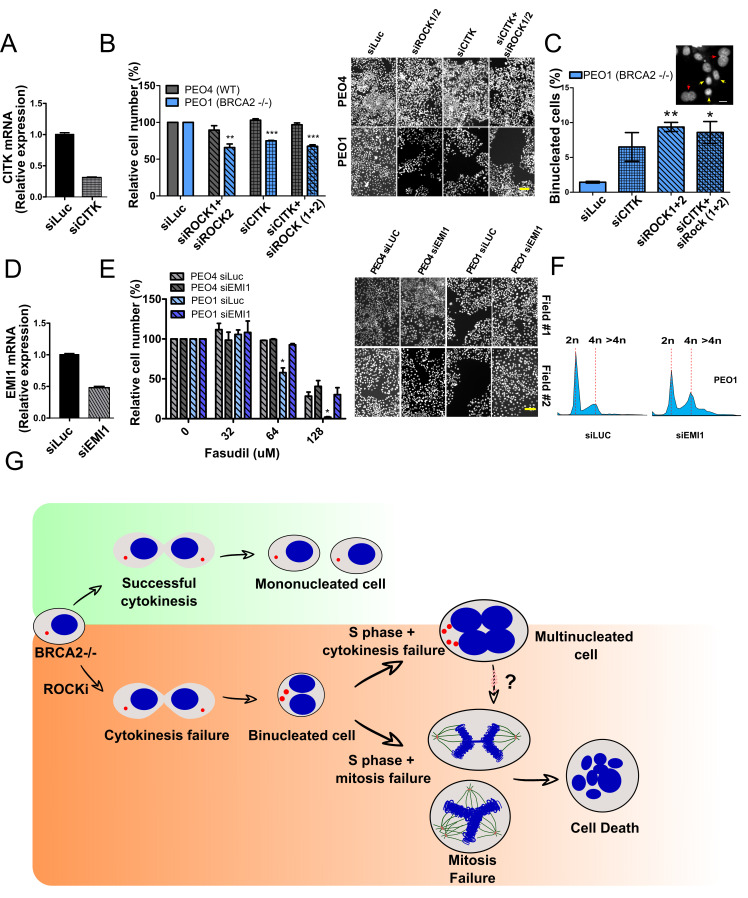


[Original Figure 8]

**Figure 2:**
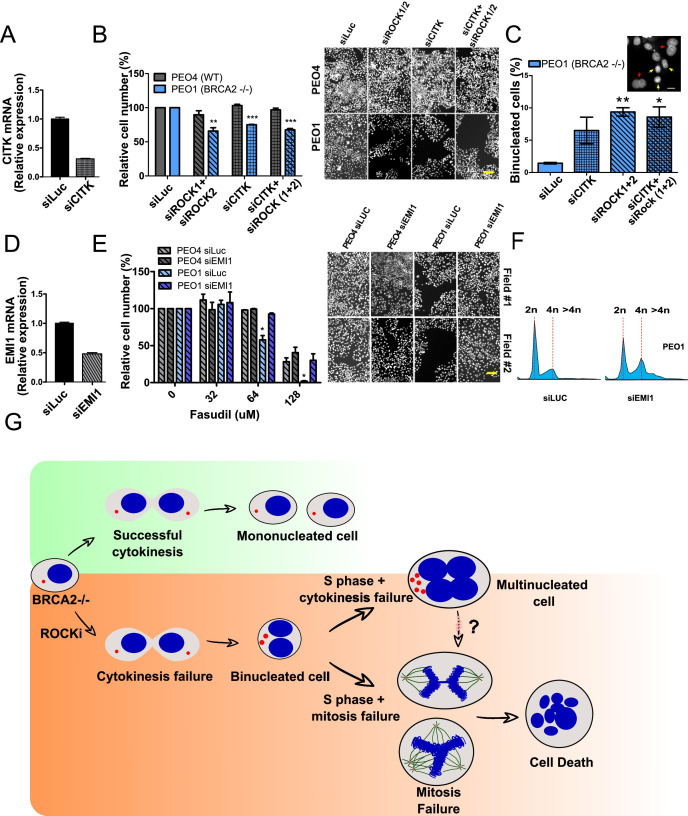


We have found 3 other mistakes/errors that need amendment:

(1) In the Results subsection "ROCK inhibition causes cytokinesis failure in BRCA2-deficient cells" there was a mistake in the citation of manuscripts covering the role of BRCA2 and ROCK in cytokinesis. The text has been modified and two missing citations were added; (2) in the Materials and methods the sequence from the shBRCA2 was missing and (3) the information regarding scales bars and their calculation needs to be added.

We apologize for the inconvenience these mistakes may have caused. The article has been corrected accordingly.

